# Enabling allogeneic therapies: CIRM‐funded strategies for immune tolerance and immune evasion

**DOI:** 10.1002/sctm.20-0079

**Published:** 2020-06-25

**Authors:** Lisa C. Kadyk, Ross M. Okamura, Sohel Talib

**Affiliations:** ^1^ California Institute for Regenerative Medicine Oakland California USA

**Keywords:** immunosuppression, cell transplantation, donor‐specific tolerance, hematopoietic chimerism, immune reconstitution, T cell, tissue engineering

## Abstract

A major goal for the field of regenerative medicine is to enable the safe and durable engraftment of allogeneic tissues and organs. In contrast to autologous therapies, allogeneic therapies can be produced for many patients, thus reducing costs and increasing availability. However, the need to overcome strong immune system barriers to engraftment poses a significant biological challenge to widespread adoption of allogeneic therapies. While the use of powerful immunosuppressant drugs has enabled the engraftment of lifesaving organ transplants, these drugs have serious side effects and often the organ is eventually rejected by the recipient immune system. Two conceptually different strategies have emerged to enable durable engraftment of allogeneic therapies in the absence of immune suppression. One strategy is to induce immune tolerance of the transplant, either by creating “mixed chimerism” in the hematopoietic system, or by retraining the immune system using modified thymic epithelial cells. The second strategy is to evade the immune system altogether, either by engineering the donor tissue to be “invisible” to the immune system, or by sequestering the donor tissue in an immune impermeable barrier. We give examples of research funded by the California Institute for Regenerative Medicine (CIRM) in each of these areas, ranging from early discovery‐stage work through clinical trials. The advancements that are being made in this area hold promise that many more patients will be able to benefit from regenerative medicine therapies in the future.

**Significance statement**

For cell and tissue therapies to become widely accessible will ultimately require the success of off‐the‐shelf allogeneic products that can be administered to patients regardless of immune compatibility with the donor tissue. Since the long‐term use of immunosuppressive drugs renders patients subject to infectious disease and other side effects, it is critical to develop alternative methods to overcome immune barriers to engraftment. The California Institute for Regenerative Medicine (CIRM) has funded multiple programs, in different disease areas and at different stages of therapeutic development, that are tackling this challenge. This article summarizes the main approaches that are being taken in this rapidly moving field and gives examples of specific programs that CIRM has funded in these different areas.

## 1 INTRODUCTION

Successful therapeutic transplantation of allogeneic cells or organs requires overcoming immunological rejection of the graft by the recipient. Although transplantation of donated tissues has saved many lives and treated many diseases, patients must undergo chronic immune suppression to prevent rejection of the life‐saving tissue, typically with a cocktail of drugs including a purine metabolism inhibitor, a calcineurin inhibitor, and steroids. These drugs are associated with cumulative side effects that increase the risk of cardiovascular disease, diabetes, hypertension, infection, cancer, and nephrotoxicity.^1^ Other immunosuppressive agents that are used clinically are monoclonal antibodies to target and eliminate reactive host lymphocytes (eg, Alemtuzumab)^2^, ^3^ or to block costimulation of T lymphocytes (eg, Abatacept).^4^ Despite this armamentarium of tools for suppressing the patient immune response, the risks of graft rejection remain high. Furthermore, when the donor tissue in question is hematopoietic stem cells (HSCs), patients are also at risk for suffering from graft vs host disease (GVHD), with side effects ranging from mild to life‐threatening. With increasing numbers of allogeneic cell‐based therapies being developed for testing in the clinic, the challenge of achieving long‐term engraftment while avoiding potentially lifelong immune suppression is being actively approached by many different groups. These strategies can be divided into two conceptually different approaches; one approach is to induce immune tolerance of the donor tissue, while the second approach is to evade the immune response altogether. The California Institute for Regenerative Medicine (CIRM) funds the development of many types of cell therapies and thus it is critical also to fund the development of methods to ensure those therapies can be safely and durably transplanted to patients. In this perspective, we briefly describe some of the strategies being taken by our awardees to address the problem of immune rejection.

## 2 APPROACHES TO INDUCE IMMUNE TOLERANCE

Table 1 summarizes multiple CIRM‐funded projects that are testing methods to induce immune tolerance, a situation in which the host immune system does not reject allogeneic transplanted tissue, and, in the case of HSC transplantation (HSCT), the donor immune cells do not attack the host tissue. Several of the clinical stage projects are using the induction of hematopoietic “mixed chimerism” to create immunological tolerance, while, in parallel, preclinical work is being done to develop ways to reprogram the immune system at the level of the thymus.

**TABLE 1:** CIRM‐funded immune tolerance approaches

Disease	Principle investigator/institution	Approach	Stage of development
Kidney disease	Sam Strober Stanford	Mixed chimerism (HLA‐matched and haploidentical donors)	Phase 1 NCT00319657
Kidney disease	Karen Smith Medeor Therapeutics	Mixed chimerism (HLA matched donors)	Phase 3 NCT03363945
Kidney disease	Everett Meyer Stanford	Mixed chimerism (haploidentical donors)	Phase 1 NCT03943238
Sickle cell disease	Joseph Rosenthal City of Hope	Mixed chimerism (haploidentical donors)	Phase 1 NCT03249831
Alpha thalassemia	Tippi MacKenzie UCSF	Mixed chimerism (haploidentical maternal donor)	Phase 1 NCT02986698
N/A	Mark Anderson UCSF	T cell reprogramming in thymus	Discovery^a^
N/A	Gay Crooks UCLA	T cell reprogramming in thymus	Discovery

Abbreviations: CIRM, California Institute for Regenerative Medicine; HLA, human leukocyte antigen.

^a^ The goal of CIRM “Discovery” stage awards is either to identify a candidate therapeutic that demonstrates reproducible disease modifying activity in a preclinical model relevant to the target indication, or to identify a medical device that demonstrates technical feasibility in meeting product design requirements and initial performance criteria.

### 2.1 Mixed chimerism

One approach to induce tolerance of an allogeneic donor graft is through generation of “mixed chimerism,” characterized by coexistence of donor and recipient blood and immune cells. Persistent mixed chimerism has been demonstrated to be required for immune tolerance, minimizing, or preventing both graft rejection by the host and GVHD.^5^, ^6^ CIRM has funded several groups using this approach for a variety of different disease indications. In one example, Dr Sam Strober and his colleagues at Stanford have enrolled and treated kidney disease patients with either human leukocyte antigen (HLA) fully matched or haploidentical (match at 3/6 HLA antigen loci) related donor combined kidney and blood stem cell transplants (Table 1). After receiving a kidney transplant, these patients were conditioned with lymphoid irradiation and antithymocyte globin and then infused with hematopoietic progenitor cell (CD34+) and T‐cell (CD3+) populations from the same donor. This study showed that HLA fully matched transplant recipients who achieved persistent mixed chimerism for at least 6 months became immunologically tolerant of the donor kidney, and it was possible to withdraw immunosuppressive medications without graft rejection or GVHD.^7^ Success of the HLA matched combined kidney and blood stem cell transplants has resulted in a progression of the Stanford Phase 1 trial to a CIRM‐funded phase 3 registrational trial conducted by Medeor Therapeutics (Table 1).

The aforementioned clinical trial led by Dr Strober also found that approximately half of patients receiving kidneys from haploidentical donors achieved stable mixed chimerism and kidney graft tolerance with reduced immune suppression (tacrolimus monotherapy), but that loss of chimerism and graft rejection occurred if immune suppression was completely withdrawn.^7^ In an effort to improve the ability to establish stable mixed chimerism in haploidentical transplants, Dr Everett Meyer is leading a CIRM‐funded trial that uses a modification of the Strober protocol, in which an infusion of autologous T regulatory cells (Tregs) is added at the time of infusion of the donor CD34+ and CD3+ cells (Table 1). It is known that Tregs help maintain immune tolerance through inhibition of self‐reactive T cells and preclinical data support the idea that adding autologous T cells to the HSCT can help maintain mixed chimerism.^8^ Success with HLA haploidentical or HLA mismatched transplants from related, living donors could open the possibility of extending this approach to transplants using HLA antigen mismatched tissues from unrelated and even deceased donors. Eventually this approach may be applicable to transplant of other organs such as hearts, lungs, and livers or even to the transplantation of cells, tissues, and organs derived from allogeneic embryonic and induced pluripotent stem cells (iPSCs).

A mixed chimerism approach is also being taken by Dr Joseph Rosenthal at City of Hope Medical Center, who is conducting a CIRM‐funded phase 1 trial to treat sickle cell disease (SCD) patients with a haploidentical HSCT using nonmyeloablative conditioning (Table 1). The concept behind this approach was inspired by earlier observations that stable mixed chimerism sometimes occurs after standard myeloablative HSCT for SCD patients, and donor chimerism between 11% and 74% was found to be curative in the absence of GVHD.^9^ The nonmyeloablative method that is used to generate mixed rather than complete chimerism is less toxic than that used for standard HSCT, and thus may be tolerated by patients with severe SCD, who would otherwise be ineligible for a transplant due to pre‐existing organ damage and other comorbidities. The use of haploidentical donors may also increase patient access to this therapy by expanding the donor pool, as many family members may be suitable matches. This trial aims to test the safety and feasibility as well as the efficacy of the approach.

Another pioneering and potentially transformative approach to developing tolerance by inducing mixed chimerism is being taken by Dr Tippi MacKenzie at UCSF, who is conducting a CIRM‐funded phase 1 trial to treat alpha thalassemia major patients in utero by transplantation of maternal HSC (Table 1). Because the fetal immune system is not fully developed, it is tolerant of maternal antigens during gestation and can accept maternal HSC without conditioning of the niche. It is not expected that full chimerism would be needed to cure the disease, based on both preclinical^10^, ^11^ and clinical^9^, ^12^ observations that donor chimerism levels as low as 10% to 20% were sufficient to cure related hemoglobin defective diseases, beta thalassemia, and SCD. In Dr MacKenzie's trial, it may be possible to cure the disease with the initial maternal HSCT, if sufficient levels of chimerism are achieved. However, even if chimerism is not sufficient to cure the disease, it may be sufficient to induce long‐term tolerance to maternal antigens, such that a postnatal “booster” transplant from the mother could be done to increase the levels of donor cells with minimal conditioning required and low risk for GVHD. Such booster transplants have been done successfully in several preclinical models.^13^

### 2.2 Immune system reprogramming

An appealing alternative to circumvent transplant rejection without immunosuppression would be to reprogram the immune system by teaching it to tolerate allogeneic transplants. Thymic epithelial cells (TECs) are critical for inducing central immune tolerance by causing the elimination of self‐reactive T cells. Thus, the ability to generate functional TECs from human pluripotent stem cells (hPSCs) may help overcome immune barriers to engraftment of other cells or tissues derived from those same stem cells. Toward that end, Dr Mark Anderson and his team at UCSF were funded by CIRM to develop a novel method for generating thymic epithelial precursors (TEPs) from hPSCs (Table 1). They showed that these hPSC‐derived TEPs are functional upon transplantation in athymic mice and acquire characteristics of mature TECs that allow them to support multistage T‐cell development and the generation of functional T cells capable of mounting allogeneic immune responses. In addition, the TEP grafts promote the formation of Tregs, which, as previously mentioned, help maintain immune tolerance through inhibition of self‐reactive T cells.^14^

A similar approach of using TEC for the purpose of inducing tolerance was taken by Dr Gay Crooks at UCLA, as part of a CIRM‐funded project in which she generated organoids composed of human fetal TEC and thymic mesenchyme cells that also are capable of supporting T‐cell differentiation (Table 1).^15^ Dr Crooks' technology has further matured to the point that her artificial thymic organoid can support efficient and scalable production of T cells in vitro from either primary HSC^16^ or reprogrammed PSC.^17^ This technology represents a significant advance in the ability to manufacture T cell‐based immunotherapies.

## 3 APPROACHES TO IMMUNE EVASION

Table 2 summarizes multiple approaches to immune evasion being developed by CIRM grantees. These can be broadly divided into two main categories: (a) engineering cells to be immunologically “incognito” by manipulating the expression of immune system proteins, and (b) developing encapsulation devices that shield the cells from the immune system. We briefly describe some of the approaches below.

**TABLE 2:** CIRM‐funded immune evasion approaches

Disease	Principle investigator/institution	Approach	Stage of development
Type 1 diabetes	Howard Foyt Viacyte, Inc.	Macroencapsulation (Encaptra device VC‐01)	Phase 1/2 NCT02239354
Type 1 diabetes	Tejal Desai UCSF	Macroencapsulation	Discovery^a^
Type 1 diabetes	Shuvo Roy UCSF	Macroencapsulation	Discovery
Heart disease	Sonja Schrepfer UCSF	Cell engineering	Discovery
Type 1 diabetes	Yang Xu UCSD	Cell engineering	Discovery
Type 1 diabetes	Alan Agulnick Viacyte, Inc.	Cell engineering	Discovery
Type 1 diabetes	Ron Evans Salk Institute	Cell engineering	Discovery
Liver failure	Tracy Grikscheit CHLA	Cell engineering	Discovery
Cancer	Lili Yang UCLA	Cell engineering	Discovery

Abbreviations: CIRM, California Institute for Regenerative Medicine; HLA, human leukocyte antigen.

^a^ The goal of CIRM “Discovery” stage awards is either to identify a candidate therapeutic that demonstrates reproducible disease modifying activity in a preclinical model relevant to the target indication, or to identify a medical device that demonstrates technical feasibility in meeting product design requirements and initial performance criteria.

### 3.1 Cell engineering to prevent immunogenicity

Genetic manipulation of hPSC can be used to enable hPSC‐derived therapeutics to evade the immune system, rather than be tolerated by it. To prevent T cell‐mediated rejection of the transplanted tissue, it is necessary to remove the HLA class I and II from that tissue. This can be done by disrupting the B2M gene, an essential component of HLA class I, and the CIITA gene, which is required for the transcription of HLA class II genes.^18^, ^19^ However, one consequence of removing the HLA antigens is that those cells are then exposed to killing by natural killer (NK) cells, which are normally inhibited from killing HLA‐positive cells. Thus, to prevent NK‐mediated killing of HLA‐deficient cells, several solutions have been explored. Universal Cells (an Astellas Company)^20^ offers a commercially available system for generating immune‐evasive cells by disrupting the B2M gene and also expressing the HLA‐E gene to suppress cytotoxicity mediated by NKG2A+ NK cells. To prevent cytotoxicity mediated by NKG2A‐NK cells, expression of HLA‐G, ligand for the inhibitory receptor KIR2DL4, has been proposed.^21^

Multiple CIRM grantees are starting to use similar cell engineering approaches to develop nonimmunogenic allogeneic cell therapies^22^ (Table 2). Dr Alan Agulnick is leading research at Viacyte to engineer nonimmunogenic embryonic stem cells (ESC), as a source of pancreatic cells for treatment of type 1 diabetes (T1D). With a similar therapeutic goal, Dr Ron Evans at the Salk Institute for Biological Sciences is developing ESC‐derived, nonimmunogenic “islet‐like organoids.” Other disease areas are also being tackled with this approach; for example, Dr Tracy Grikscheit of Children's Hospital Los Angeles (CHLA) plans to engineer human iPSC to be nonimmunogenic before deriving hepatic progenitors to be tested in liver disease models, and Dr Lili Yang plans to engineer nonimmunogenic HSC to derive invariant natural killer T (iNKT) cells for use as a cancer therapeutic.

In a variation of the above approaches, Dr Sonja Schrepfer at UCSF was funded by CIRM to couple HLA class I and II disruption with CD47 overexpression in human iPSC, followed by derivation of cardiomyocytes, smooth muscle, and endothelial cells that were used to create a hypoimmunogenic cardiac patch. In preclinical testing, this patch evaded immune rejection after transplantation into fully major histocompatibility complex (MHC) mismatched allogeneic recipients.^23^ The results indicate that CD47 expression may prevent NK cell responses, consistent with an association between CD47 expression and protection from NK cell‐mediated cytotoxicity that was described previously in a study of squamous cell carcinoma lines.^24^ As an added benefit, overexpression of CD47 prevents phagocytosis by macrophages.^25^

Naturally occurring suppressors of the immune system have also suggested other ways to engineer cellular therapies to prevent recognition by the host immune system. CIRM‐funded research led by Dr Yang Xu at UCSD has found that the expression of checkpoint molecules CTLA4 (CTLA4‐Ig) and PD‐L1 in human ESC line HUES3 was sufficient to prevent rejection of differentiated tissues (teratomas, fibroblasts, and cardiomyocytes) in mice bearing humanized immune systems.^26^ CIRM has continued to sponsor Dr Xu's research, extending its application toward protection of allogeneic hPSC‐derived beta cells, which could ultimately be used for treating T1D.

We note that the above strategies, should they be successful, would prevent the immune system from recognizing donor tissue even if became infected or cancerous. Therefore, many researchers also include an inducible “kill switch” such as iCasp9^27^ or herpes simplex virus thymidine kinase^28^ so it would be possible to destroy the engrafted cells should the need arise.

### 3.2 Encapsulation devices

Among the strategies to avoid the immune system, encapsulation is perhaps conceptually the simplest way to protect a cellular therapeutic while at the same time being the most limited in its application. Both microencapsulation devices, in which relatively small numbers of cells are embedded in matrices such as alginate, polyethylene glycol (PEG), or gelatin, and macroencapsulation devices (>1 mm), capable of containing larger amounts of cells, have arisen as viable approaches to evading the immune system.^29^, ^30^ The requirements for these encapsulation modalities are generally (a) that they are biostable within the given implantation site, (b) that they permit sufficient nutrient flow into the capsule as well as release of secreted molecules out of the capsule, and (c) that they do not induce a foreign body response and fibrotic encapsulation. Since encapsulation shields the therapeutic from any direct physical interaction with the immune system and other body tissues, this type of therapy is suited for enzyme or hormone replacement therapies, rather than regeneration of large amounts of tissues or organs. One disease for which this strategy seems appropriate is T1D, where regulation of blood glucose by insulin production from pancreatic beta cells is impaired. Encapsulation of allogeneic insulin‐producing pancreatic beta cells within semipermeable membranes would allow for the diffusion of insulin from the cell therapeutic into the blood system, while also allowing for the diffusion of oxygen and glucose into the capsule to support the viability of the cells within.

In this area, CIRM has supported the development of ViaCyte's Encaptra macroencapsulation device for the treatment of T1D with hESC‐derived pancreatic precursor cells (PEC‐01 cells).^31^ The Encaptra device is a single membrane planar pouch composed of expanded polytetrafluoroethylene and was designed to protect PEC‐01 cells from immune responses while allowing for insulin release in response to blood glucose levels. The device is implanted subcutaneously which allows for the device to be retrieved if necessary, an important safety consideration. The immune‐isolating Encaptra device VC‐01 (Table 1), as well as a variant that allows direct vascularization and so requires use of immunosuppression, has been used in CIRM‐funded clinical trials. Recent results from the clinical trial (NCT03163511) using the direct vascularization device have shown evidence of circulating C‐peptide, a biomarker of insulin production, in T1D patients who previously had no detectable C‐peptide.^32^ Although that device is not immune evasive, the demonstration that the PEC‐01 cells differentiate and become functional in patients further emphasizes the importance of developing an immune evasive strategy to support maintenance of these cells in the absence of immune suppression.

Dr Tejal Desai at UCSF has also received CIRM funding to develop a macroencapsulation technology for T1D based on flexible nanoporous thin films designed to support the long‐term viability and function of allogeneic human ESC‐derived insulin producing beta cells. Dr Desai has placed an emphasis on the composition of her device being able to avoid a scar‐forming foreign body response that would affect the viability and function of the beta cells. Encellin, a San Francisco‐based biotechnology company, has licensed Dr Desai's technology for commercialization.

Dr Shuvo Roy, also at UCSF, is developing a CIRM‐funded intravascularly implanted macroencapsulation device, a bioartificial pancreas (iBAP) for T1D, that is conceptually different from the Viacyte and Encellin devices. Instead of relying on the proximity of surrounding vasculature to allow exchange of oxygen, glucose, and insulin between the encapsulated cells and the blood system, the iBAP is implanted directly in‐line with a blood vessel to provide enhanced kinetics of glucose sensing and insulin secretion while still isolating the allogeneic human stem cell derived pancreatic beta cells from the immune system using a silicon nanopore membrane.^33^

## 4 CONCLUSION

The ability to use allogeneic cells and tissues for regenerative medicine will be critical to enable large‐scale, reproducible manufacturing that will both increase availability of the treatment and manage the cost of the products. Thus, as more regenerative cell therapies enter the clinic and begin to show efficacy in patients, it is critical, in parallel, to develop safe and easily implemented methods to overcome immunological barriers in order to facilitate durable engraftment of transplanted allogeneic tissues. Herein, we have described some of the approaches being taken by CIRM grantees to enable successful engraftment of allogeneic tissues, which are a small subset of the many efforts being made in this field. For example, other methods being used to induce immune tolerance include adoptive transfer of cell types such as Tregs, tolerogenic dendritic cells, and mesenchymal stromal cells.^34^, ^35^, ^36^ In addition, there are many groups working on different ways to encapsulate or otherwise shield grafted cells from the immune response,^29^ and to create immune evasive cells.^37^ Although many of these approaches are still relatively new and have not yet been implemented clinically, there is great promise that improved methods for enabling engraftment of allogeneic cells, organs, or tissues will enable much broader use of regenerative therapies as they are developed.

## CONFLICT OF INTEREST

The authors declared no potential conflicts of interest.

## AUTHOR CONTRIBUTIONS

L.C.K., R.M.O., S.T.: conception and design, manuscript writing and final approval of manuscript.
